# Supplementary material for: Cladribine Reduces Trans-Endothelial Migration of Memory T Cells across an In Vitro Blood–Brain Barrier
Source: J Clin Med. 2022 Oct 12;11(20):6006. doi: 10.3390/jcm11206006 (PMC9604596; doi:10.3390/jcm11206006)
Supplement: Supplementary file 1 [file jcm-11-06006-s001.zip › jcm-1944059-supplementary.pdf]

Supplementary Material:

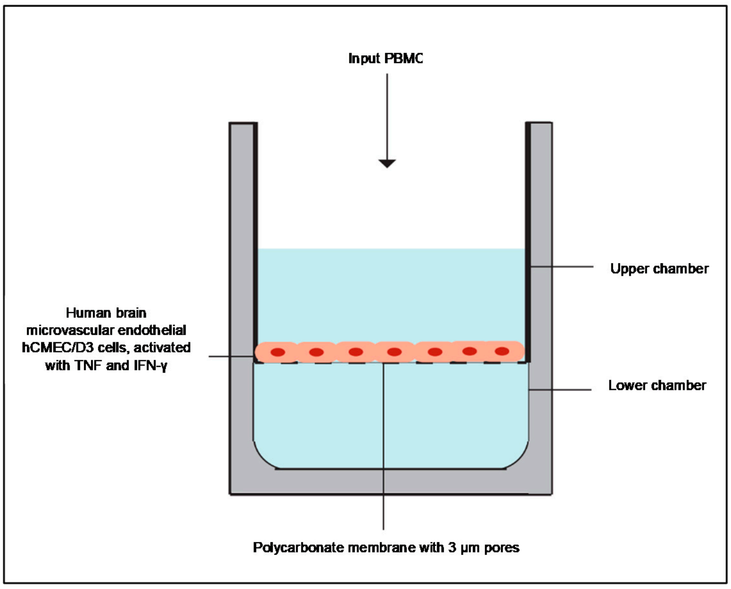

Supplementary Figure S1. Experimental setup of in vitro BBB model used for transmigration assays. The two chambers were separated by a porous membrane to represent the peripheral blood (upper chamber) and the CNS blood (lower chamber). The membrane between compartments consisted of 3 µm pores and was coated in collagen, a component of the basement membrane within the BBB. Collagen was overlaid with human brain microvascular endothelial cells (HBEC), sourced from the hCMEC/D3 cell line, which simulated the endothelial cell barrier within the BBB. Additionally, TNF and IFN-γ were used to activate the BBB thereby mimicking a disease state. PBMC were isolated from the blood of untreated MS patients, cladribine-treated MS patients and healthy controls and loaded onto the top compartment to be left to migrate overnight. PBMC, peripheral blood mononuclear cells.

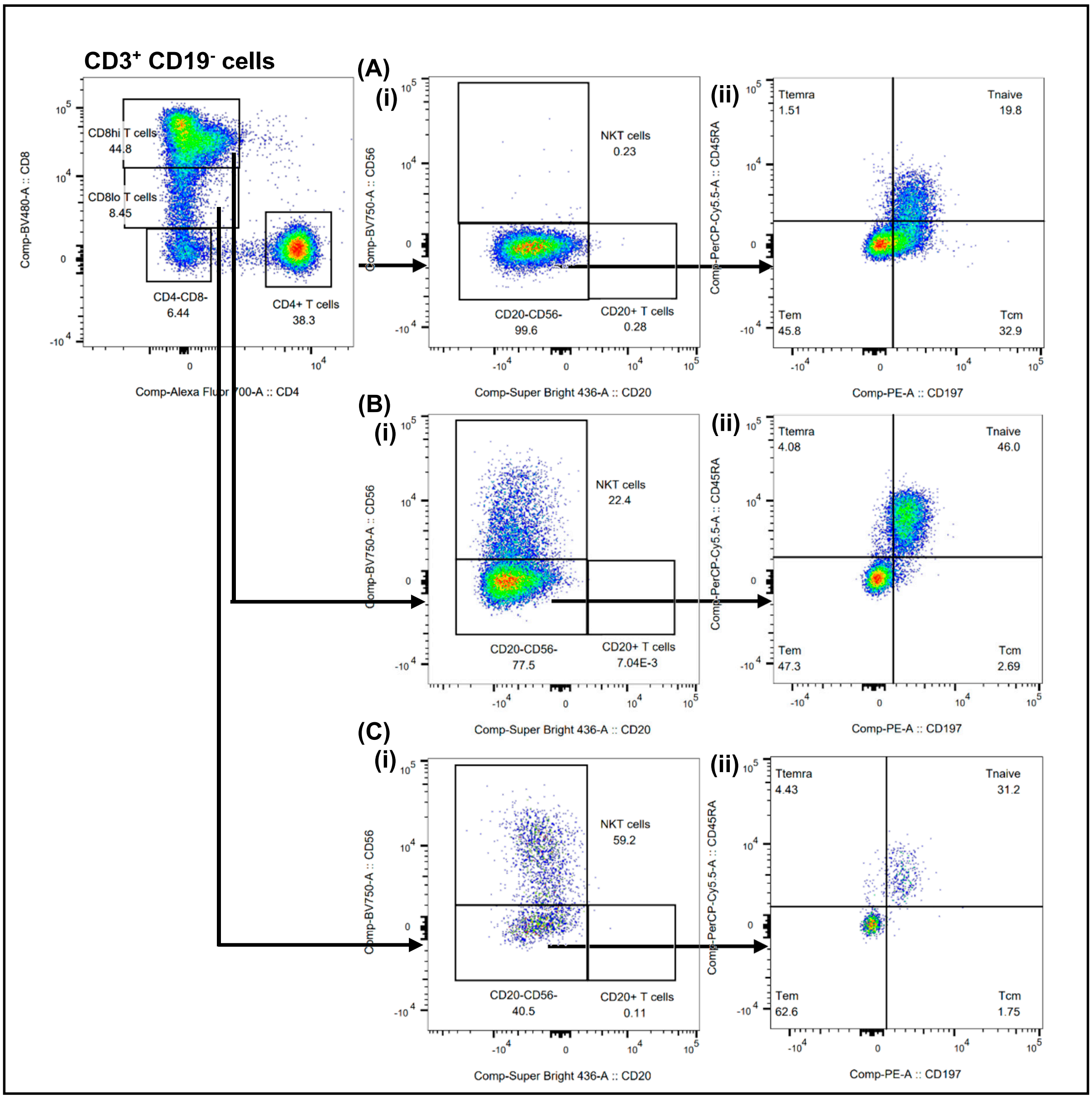

Supplementary Figure S2. T cell flow cytometry immunophenotyping gating strategy. Distinct CD4^{+}, CD8^{high} and CD8^{low} T cell populations were identified from CD19^{-}CD3^{+} T cells. Within **(A)** CD4^{+}, **(B)** CD8^{high} and **(C)** CD8^{low} T cell populations, gating strategy progressed from (i) to (ii) as indicated by arrows to identify **(i)** NKT cells (CD56^{+}CD20^{-}), CD20^{+} T cells (CD56^{-}CD20^{+}) and **(ii)** T^{TEMRA} (CD45RA^{+}CD197^{-}), T^{naïve} (CD45RA^{+}CD197^{+}), T^{CM} (CD45RA^{-}CD197^{+}) and T^{EM} (CD45RA^{-}CD197^{-}) cells. NKT, natural killer T cells; T^{TEMRA}, terminally differentiated effector memory cells re-expressing CD45RA T cells; T^{naive}, naïve T cells; T^{CM}, central memory T cells; T^{EM}, effector memory T cells.

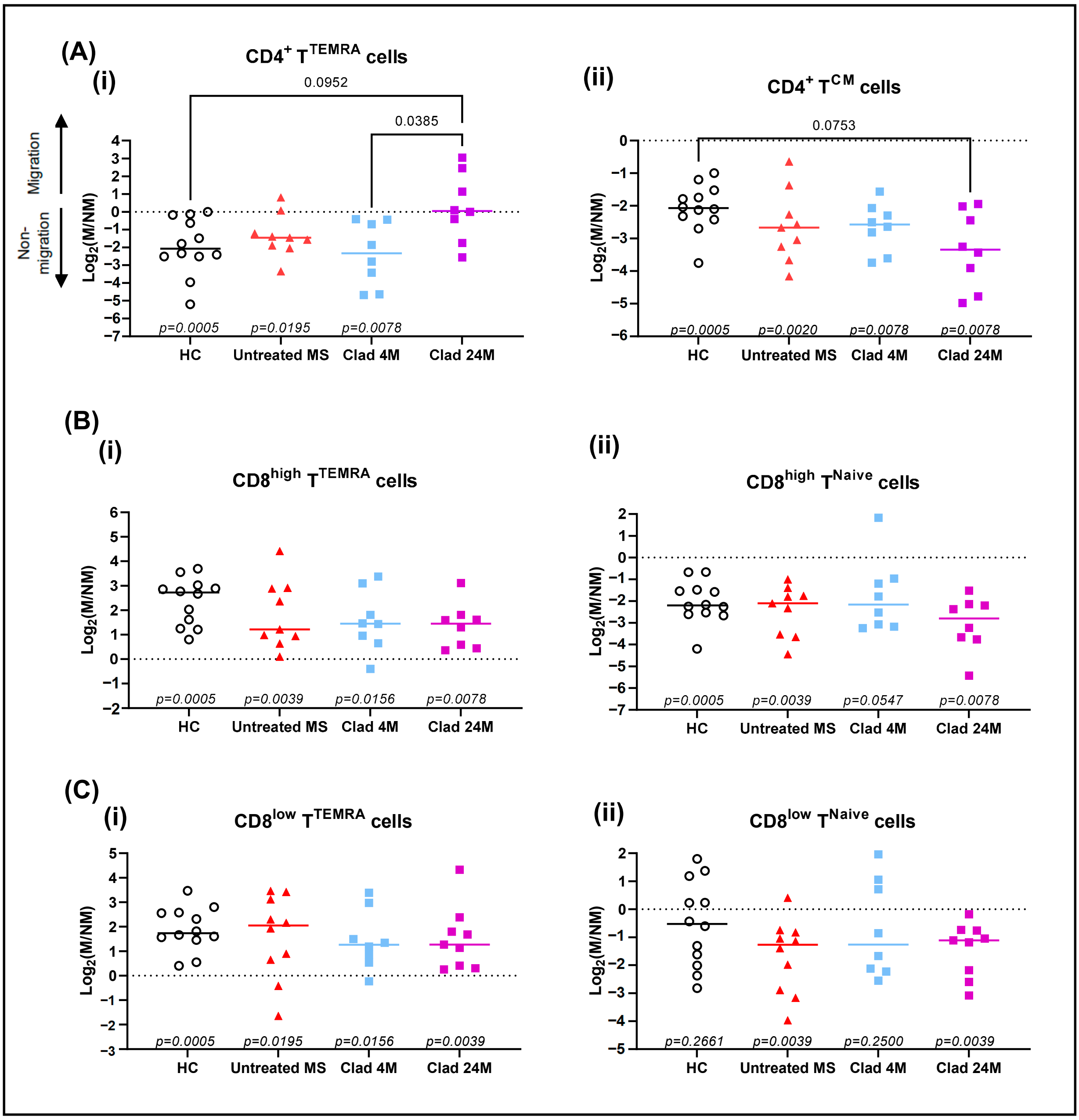

Supplementary Figure S3. The trans-endothelial migration of CD4+ and CD8+ T cells subsets. PBMCs were isolated from healthy controls (n=12), untreated MS (n=9) and cladribine-treated MS patients at 4 months (n=8) and 24 months (n=8) post initial dose. PBMC were then added to an *in vitro* model of the stimulated BBB and left to migrate overnight. The logarithm of 2-fold change of migrated to non-migrated cells was calculated. **(A)** The migratory capacity of **(i)** CD4^{+} T^{TEMRA} cells and **(ii)** CD4+ T^{CM} cells. **(B)** The migratory capacity of **(i)** CD8^{high} T^{TEMRA} cells and **(ii)** The CD8^{high} T^{Naive} cells. **(C)** The migratory capacity of **(i)** CD8^{low} T^{TEMRA} cells and **(ii)** CD8^{low} T^{Naive} cells. In-group analysis performing using one sample Wilcoxon matched-pairs signed rank test with Pratt method with p-values ≤0.1 displayed at the bottom of each graph. Between-group analysis performed using Kruskal-Wallis with Dunn's multiple comparisons test. Median shown. NM, non-migrating; M, migrating; T^{TEMRA}, terminally differentiated effector memory T cells re-expressing expressing CD45RA T cells; T^{CM}, central memory T cells; T^{EM}, effector memory T cells; T^{Naive}, naïve T cells; HC healthy controls; MS, multiple sclerosis; BBB, blood-brain barrier; Clad, cladribine; 4M, 4 months post-Clad; 24M, 24 months post-Clad; PBMC, peripheral blood mononuclear cells.

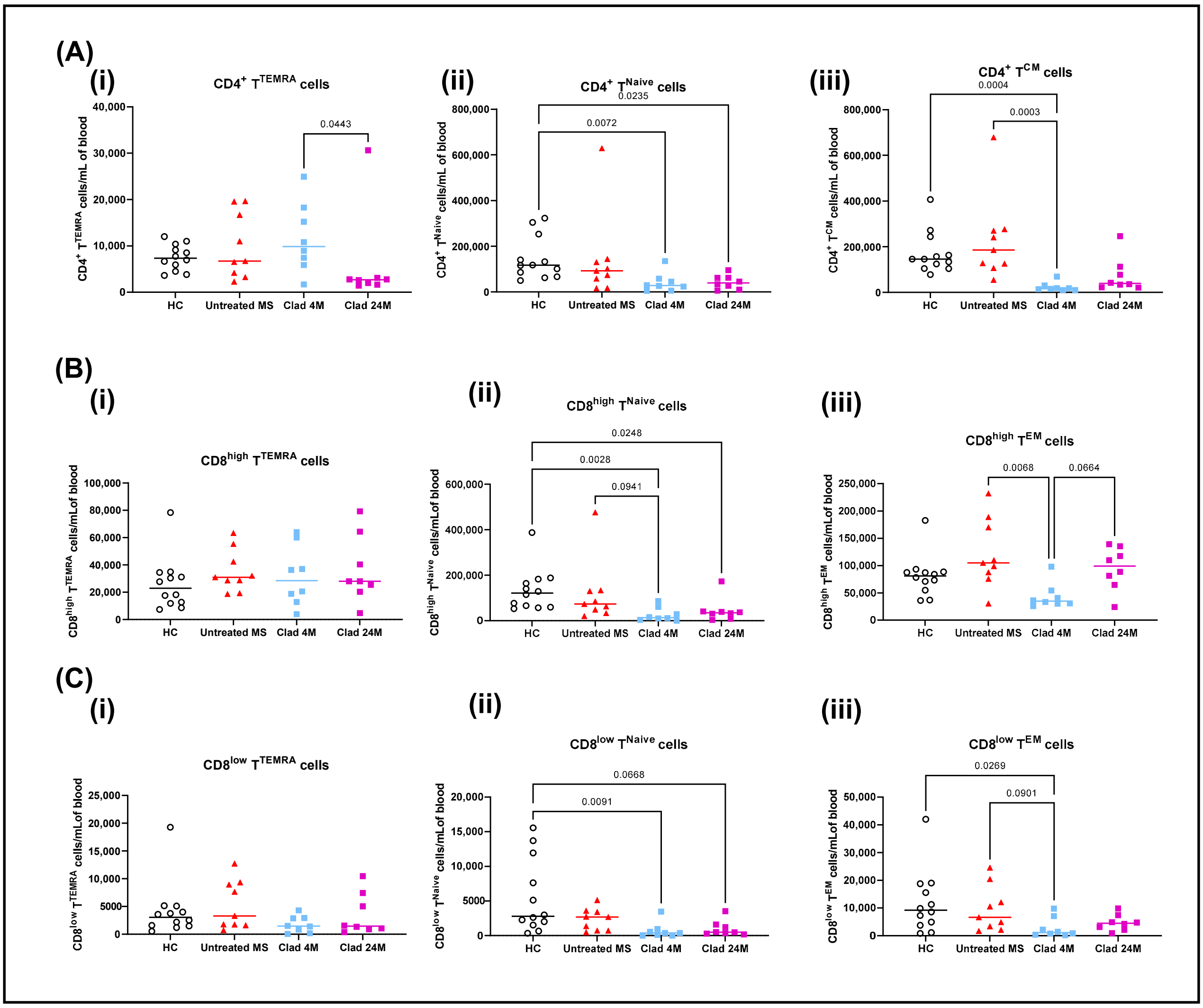

Supplementary Figure S4. The peripheral blood concentration of CD4+ and CD8+ T cells subsets. Blood was sampled from healthy controls (n=12), untreated MS (n=9) and cladribine-treated MS patients at 4 months (n=8) and 24 months (n=8) post initial dose. Upon sample collection, fresh PBMC were immediately isolated and phenotyped. Each T cell subset was gated on to calculate the proportion of cells multiplied by the cell count and shown as cells per mL of blood. **(A)** The peripheral blood concentration of **(i)** CD4+ T^{TEMRA} cells **(ii)** CD4+ T^{Naive} cells and **(iii)** CD4+ T^{CM} cells. **(B)** The peripheral blood concentration of **(i)** $CD8^{high}$ T^{TEMRA} cells **(ii)** $CD8^{high}$ T^{Naive} cells and **(iii)** $CD8^{high}$ T^{EM} cells. **(C)** The peripheral blood concentration of **(i)** $CD8^{low}$ T^{TEMRA} cells **(ii)** $CD8^{low}$ T^{Naive} cells and **(iii)** $CD8^{low}$ T^{EM} cells. Kruskal-Wallis with Dunn's multiple comparisons test. Median and p-values ≤0.1 are shown. T^{TEMRA}, terminally differentiated effector memory cells re-expressing CD45RA T cells; T^{Naive}, naïve T cells; T^{CM}, central memory T cells; T^{EM}, effector memory T cells; HC, healthy controls; MS, multiple sclerosis; Clad, cladribine; 4M, 4 months post-Clad; 24M, 24 months post-Clad; PBMC, peripheral blood mononuclear cells.
